# 3D quantification of changes in pancreatic islets in mouse models of diabetes type I and II

**DOI:** 10.1242/dmm.045351

**Published:** 2020-12-18

**Authors:** Urmas Roostalu, Jacob Lercke Skytte, Casper Gravesen Salinas, Thomas Klein, Niels Vrang, Jacob Jelsing, Jacob Hecksher-Sørensen

**Affiliations:** 1 Gubra ApS, 2970 Hørsholm, Denmark; 2 Department of CardioMetabolic, Diseases, Boehringer Ingelheim Pharma GmbH & Co. KG, 88400 Biberach, Germany

**Keywords:** Light-sheet fluorescence microscopy, Tissue clearing, Beta cells, Insulin, Inflammation

## Abstract

Diabetes is characterized by rising levels of blood glucose and is often associated with a progressive loss of insulin-producing beta cells. Recent studies have demonstrated that it is possible to regenerate new beta cells through proliferation of existing beta cells or trans-differentiation of other cell types into beta cells, raising hope that diabetes can be cured through restoration of functional beta cell mass. Efficient quantification of beta cell mass and islet characteristics is needed to enhance drug discovery for diabetes. Here, we report a 3D quantitative imaging platform for unbiased evaluation of changes in islets in mouse models of type I and II diabetes. To determine whether the method can detect pharmacologically induced changes in beta cell volume, mice were treated for 14 days with either vehicle or the insulin receptor antagonist S961 (2.4 nmol/day) using osmotic minipumps. Mice treated with S961 displayed increased blood glucose and insulin levels. Light-sheet imaging of insulin and Ki67 (also known as Mki67)-immunostained pancreata revealed a 43% increase in beta cell volume and 21% increase in islet number. S961 treatment resulted in an increase in islets positive for the cell proliferation marker Ki67, suggesting that proliferation of existing beta cells underlies the expansion of total beta cell volume. Using light-sheet imaging of a non-obese diabetic mouse model of type I diabetes, we also characterized the infiltration of CD45 (also known as PTPRC)-labeled leukocytes in islets. At 14 weeks, 40% of the small islets, but more than 80% of large islets, showed leukocyte infiltration. These results demonstrate how quantitative light-sheet imaging can capture changes in individual islets to help pharmacological research in diabetes.

## Abstract (precis)

**Summary:** We report a 3D quantitative imaging platform (using light-sheet fluorescence microscopy) that can reveal changes in beta cell volume and proliferation, and leukocyte infiltration in pancreata in mouse models of diabetes.

## INTRODUCTION

Diabetes is a life-threatening chronic disease that arises when the body is unable to regulate blood glucose homeostasis (Kitabchi et al., 2009). The hormone insulin has a central role in the regulation of blood glucose, and the main causes of diabetes are loss of insulin-producing beta cells (type I diabetes) and peripheral insulin resistance, whereby beta cells fail to meet the required insulin demand (type II diabetes). For almost a century, hormone replacement therapy using insulin has been the preferred choice for treatment of diabetes (Joshi et al., 2007). However, in recent years, there has been an increased focus on finding drugs or therapies that can either prevent the destruction of beta cells or restore functional beta cell mass and thereby cure the disease (Ben-Othman et al., 2017; Li et al., 2017; Zhou et al., 2008). Consequently, the development of novel tools and models that can reliably detect changes in beta cell mass and health is needed.

Stereology and optical projection tomography (OPT) have traditionally been considered the state-of-art methods to measure preclinical changes in beta cell mass in both 2D (Bock et al., 2003; Cucak et al., 2016; Dalbøge et al., 2013, 2014; Hansen et al., 2014; Paulsen et al., 2010) and 3D (Alanentalo et al., 2007, 2008) . Although both methods provide similar results in beta cell mass, the 3D approach offers additional information regarding islet composition because it enables volumetric quantification of individual islets (Alanentalo et al., 2007). In models of type I diabetes, the immune cells infiltrate the islets and kill the insulin-producing beta cells (Gianani and Eisenbarth, 2005). However, the progression of the disease is highly heterogeneous, and the amount of infiltration can vary greatly between two neighboring islets (Alanentalo et al., 2010; Mathews et al., 2015). Consequently, the ability to look at the whole pancreas in its entirety is important in order to fully understand the underlying pathology.

This study was aimed at developing imaging technologies for better understanding of diabetes and for more efficient drug discovery. We used 3D light-sheet imaging and quantitative image analysis to characterize changes in beta cell volume and proliferation in an inducible mouse model of type II diabetes, relying on chronic insulin antagonist administration in obese mice, and used 3D imaging in a non-obese diabetic (NOD) mouse model to chart leukocyte infiltration in pancreata.

## RESULTS

### Pipeline for 3D imaging of beta cell volume and proliferation in the entire pancreata

**Fig. 1.**
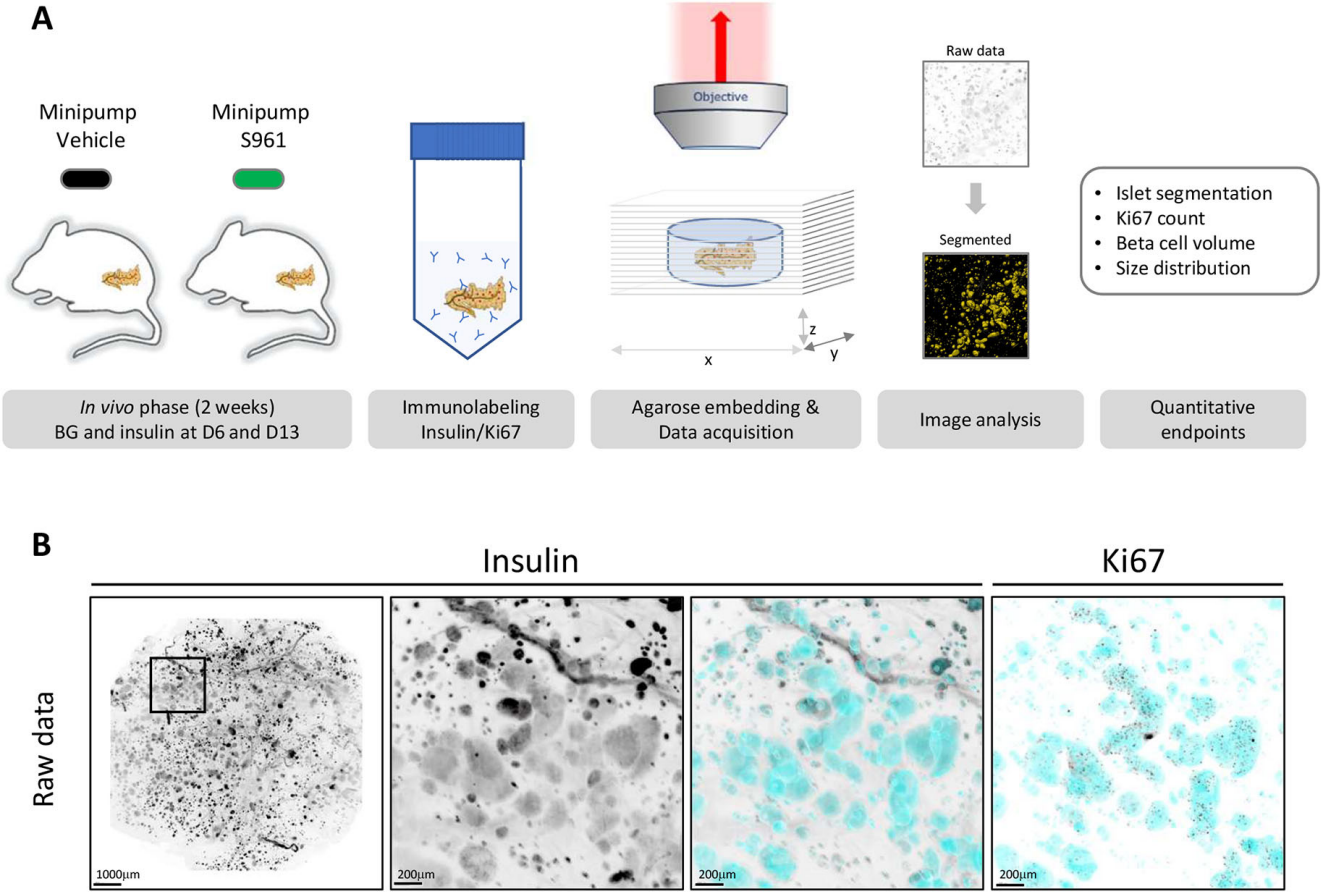
**Schematic representation of the study workflow.** (A) C57BL/6JRj diet-induced obese (DIO) mice were implanted with minipumps containing either vehicle or the insulin receptor antagonist S961. Plasma glucose and insulin were measured at day 6 and day 13. After 2 weeks, the pancreata were isolated and stained with antibodies against insulin and Ki67. For scanning, the pancreas was embedded in agarose and cleared. The insulin channel was used as a reference to segment the beta cell volume, allowing quantification of the total number of insulin-positive islets, total beta cell volume and total number of Ki67-positive beta cells. (B) The entire pancreas was scanned using light-sheet fluorescence microscopy. The insulin channel was used to segment the beta cell volume (cyan), allowing quantification of numbers and volumes of insulin-positive beta cells. The Ki67 channel was used to count the number of proliferating beta cells. Scale bars: 1000 µm (low magnification); 200 µm (high magnification).

Fast volumetric analysis of changes in islet morphology and molecular characteristics in an entire pancreas would enable more efficient preclinical pharmacological research. Here, we set out to establish a light-sheet fluorescence microscopy 3D imaging platform with sufficient sensitivity to detect changes in beta cell volume in a mouse model of diabetes, focusing first on type II diabetes. For this purpose, mice were treated for 2 weeks with either vehicle or the insulin receptor antagonist S961 (Fig. 1A). During the *in vivo* phase, blood glucose and plasma insulin levels were measured at day 6 and day 13, and an oral glucose tolerance test (OGTT) was performed at day 13. At day 14, the mice were killed, and the pancreata removed and immunolabeled with antibodies against insulin and Ki67 (also known as Mki67) using a modified version of iDISCO (Renier et al., 2014). Prior to clearing, the whole pancreas was embedded in low-melting-point agarose using the chamber of a 10 ml syringe, from which the tip had been removed, as a mold. The resulting agarose block that was cleared in dibenzyl ether had a diameter of ∼12 mm, which allows the entire pancreas to be scanned in one tile. This minimized overall scanning time and enabled us to maintain the same imaging settings for all samples (i.e. position in laser lines), but, as a result, the morphological features, such as head and tail, could no longer be distinguished from each other. The scanned images were processed for image analysis using the insulin channel to segment individual islets (Movie 1). This segmentation was subsequently used to quantify the total beta cell volume, the total number of insulin-positive islets and the total number of Ki67-positive beta cells (Fig. 1B; Movie 2).

### S961 treatment leads to impaired glucose handling

**Fig. 2.**
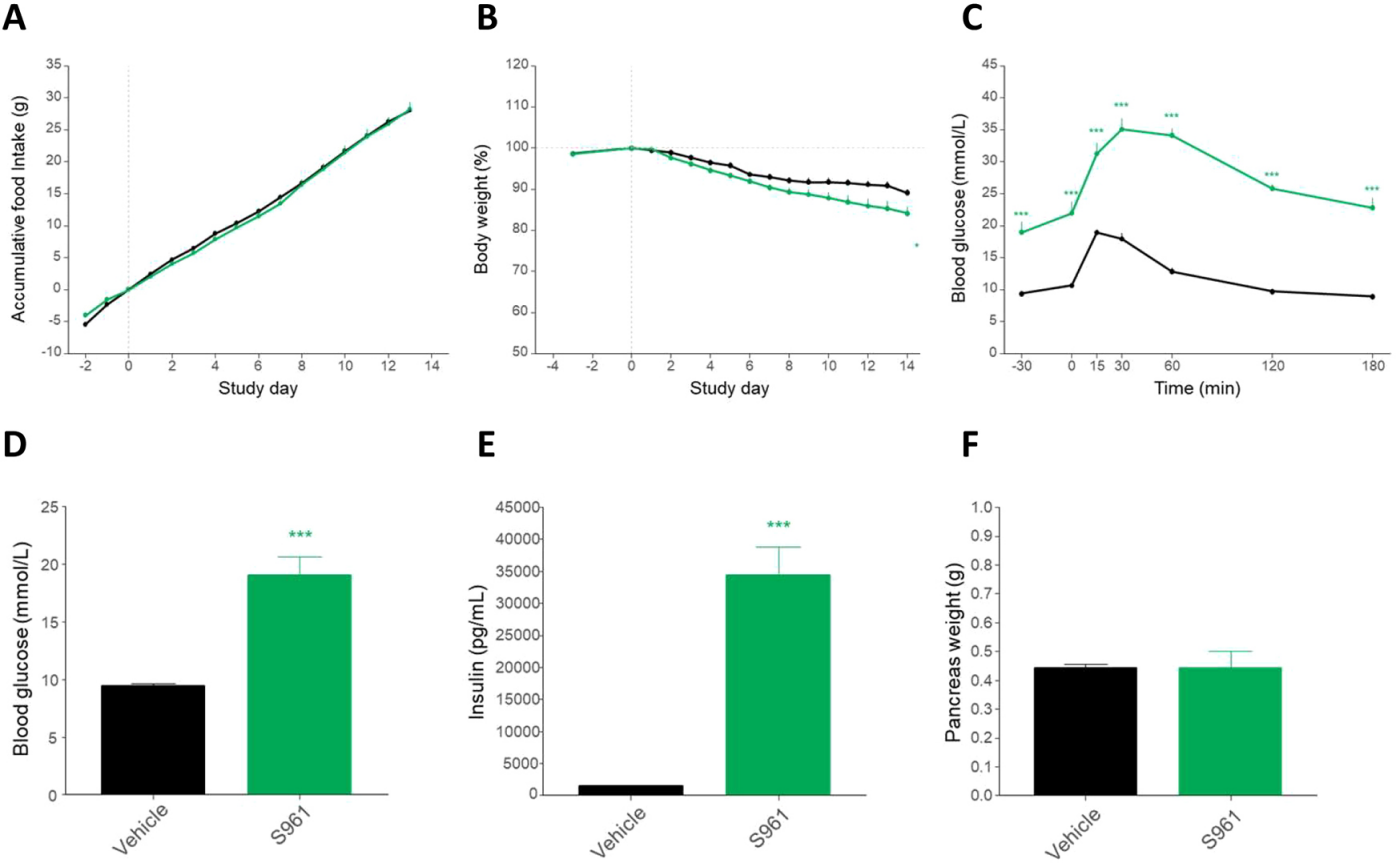
***In vivo* measurements in mice** **treated** **with either vehicle or S961.** (A) Accumulated food intake during the 2-week study was similar in the two groups. (B) The S961 mice lost slightly more weight than the vehicle mice but the difference was only significant at day 14. (C) Oral glucose tolerance test identified significantly increased blood glucose in the S961 group compared to the vehicle group. (D) At day 13, the mean plasma glucose levels were 9.50±0.22 mmol/l in the vehicle group and 20.02±1.48 mmol/l in the S961 group. (E) The mean plasma insulin levels were 1450±96.1 pg/ml in the vehicle group and 34400±884 pg/ml in the S961 group. (F) Total pancreas weight did not differ significantly between the two groups. For statistical analysis, unpaired Student's *t*-tests were used. ***P<0.001, S961 compared to vehicle. Error bars represent s.e.m.

The insulin receptor antagonist S961 is a single-chain peptide of 43 amino acids that binds with high affinity to the insulin receptor, but without activating it (Schäffer et al., 2008). Blocking of insulin receptor signaling using S961 has previously been shown to rapidly induce proliferation of existing beta cells (Dumayne et al., 2020; Jiao et al., 2014; Yi et al., 2013). Throughout the study, all mice had free access to high-calorie food, and the two groups consumed the same amount of food (Fig. 2A). Owing to the surgery required for implantation of the minipumps, a small decrease in body weight was observed in both groups. However, the weight loss was more pronounced in the S961 group (Fig. 2B), corresponding to the catabolic phenotype expected from impaired insulin signaling. After 13 days of treatment, the mice were subjected to an OGTT (Fig. 2C). At all points measured, the S961-treated mice displayed significantly higher blood glucose levels, and the return to baseline was delayed. Similarly, both plasma insulin levels and fasting blood glucose were markedly increased in the S961-treated mice at day 6 and day 13 (8.15 mmol/l and 17.1 mmol/l in the vehicle and S961-treated group, respectively; Fig. 2D,E). At termination, the whole pancreas was removed and weighed. No difference in pancreas weight was observed between the two groups (Fig. 2F). Taken together, these observations indicate that S961 effectively blocked insulin signaling, leading to a diabetic phenotype.

### Increased beta cell volume in S961-treated mice

**Fig. 3.**
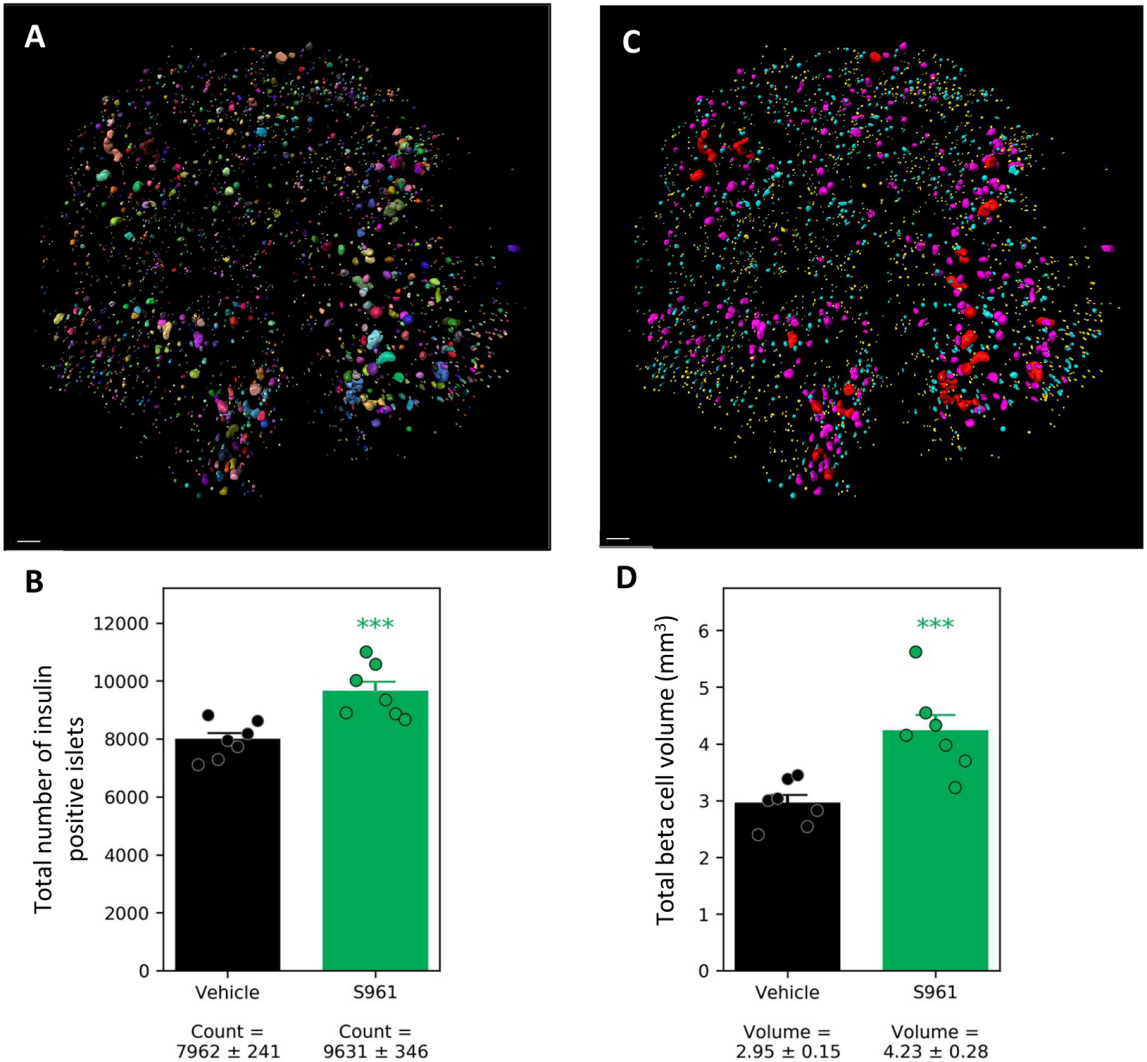
**Increased beta cell volume and number of insulin-positive islets following S961** **treatment.** (A) Using the insulin signal, each islet was assigned a unique ID and a volume, making it possible to calculate the total number of insulin-positive islets and the total beta cell volume. (B) Total number of insulin-positive islets in vehicle- and S961-treated mice. (C) The same pancreas as shown in A, color coded for size distribution. The insulin-positive islets were allocated into four bins: small (yellow; 25-170×1000 µm^3^), medium (cyan; 170-1100×1000 µm^3^), large (magenta; 1100-7500×1000 µm^3^) and very large (red; 7500-50000×1000 µm^3^). (D) Total mean beta cell volume in the study groups. Individual data points are indicated. For statistical analysis, we used unpaired Student's *t*-test. ***P<0.001, S961 compared to vehicle. Error bars represent s.e.m. Scale bars: 500 µm.

In order to determine the effect of the metabolic changes on beta cell volume, the pancreata from all mice were immunolabeled with antibodies against insulin and the cell proliferation marker Ki67 using a modified iDISCO protocol (Renier et al., 2014). The insulin-segmented signal (Fig. 3A) also allowed quantification of the total number of insulin-positive islets and beta cell volume in each pancreas. A low-size cut-off of 25×1000 µm^3^ for islets was implemented to avoid false-positive signal that may result, for example, from the non-specific presence of fluorophore in the tissue (from fluorophore-conjugated secondary antibody). The mean number of insulin-positive islets was 7962±241 in the vehicle mice and 9631±346 in the S961-treated mice, corresponding to a 21% increase in the S961 group (Fig. 3B). Similarly, the mean beta cell volume in the pancreas was 2.95±0.15 mm^3^ in the vehicle group and 4.23±0.28 mm^3^ in the S961 group, corresponding to a 43% increase in the S961 group (Fig. 3C,D). Hence, S961 treatment resulted in a significant (*P*≤0.001) increase in both islet count and volume.

### Islets of all sizes are affected by S961 treatment

**Fig. 4.**
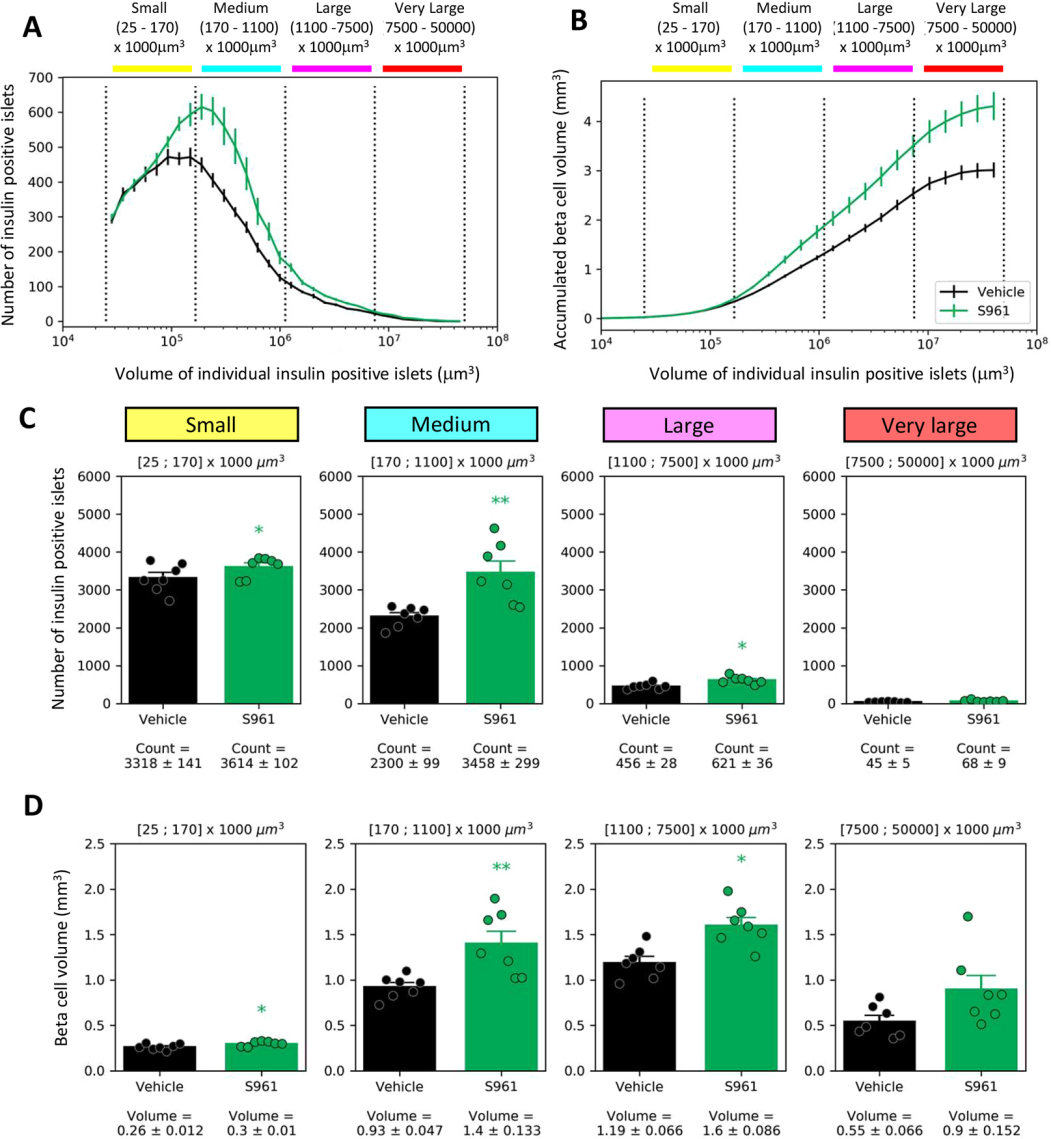
**S961** **treatment** **response in different islet size categories:** (A) Size distribution curve of islets. Size categories as indicated in the Fig. 3 legend. (B) Relative contribution of islets in different size categories to the total islet volume. Islets across all size categories contribute to the overall increase in total beta cell volume. (C) Quantification of the number of small (yellow), medium (cyan), large (magenta) and very large (red) insulin-positive islets in vehicle- and S961-treated mice. In all four bins, there is an increase in the number of insulin-positive islets following S961 treatment. (D) Quantification of the beta cell volume in the small (yellow), medium (cyan), large (magenta) and very large (red) islets in vehicle- and S961-treated mice. Individual data points are indicated. In C and D, both sets of data were separately investigated using 2×4 mixed ANOVA, and in follow-up tests on the treatment effect within each size category, the two measures were investigated in a multivariate manner (owing to correlation) by Hotelling’s T-squared test. *P<0.05, **P<0.01; S961 compared to vehicle. Error bars represent s.e.m.

Because there is considerable interest in defining the heterogeneity of islets and the response of different size islets to diabetes and drug treatment (Aizawa et al., 2001; Baetens et al., 1979; Lehmann et al., 2007; Saito et al., 1978), we allocated the segmented islets into four size bins: small (yellow; 25-170×1000 µm^3^), medium (cyan; 170-1100×1000 µm^3^), large (magenta; 1100-7500×1000 µm^3^) and very large (red; 7500-50000×1000 µm^3^), and charted the location of these categories in all pancreata (Fig. 3C; Fig. S1). When looking at the accumulated beta cell volume as a function of individual insulin-positive islet size, it is evident that there was a continuous increase in the total beta cell volume following S961 treatment across the islet size categories (Fig. 4A,B).

To characterize the changes in more detail, we looked at the islet size categories separately (Fig. 4C). In the small insulin-positive islet category, our analysis counted 3318±141 islets in the vehicle group and 3614±102 islets in the S961 group, which corresponds to 8.9% increase in the S961 group. For the medium islet category, the numbers were 2300±99 in the vehicle group and 3458±299 in the S961 group, representing a 50.3% increase in the S961 group (*P*≤0.05). In the large islet category, there were 456±28 islets in the vehicle group and 621±36 in the S961 group, corresponding to a 36.1% increase in the S961 group. In the very large islet category, there were 45±5 islets in the vehicle group and 68±9 in the S961 group, corresponding to a 51.1% increase in the S961 group. Statistical analysis of the islet count data revealed significant main effects of both treatment [*F*(1,12)=18.37, *P*=0.0011] and size category [*F*(3,36)=356.93, *P*<0.001], and their two-way interaction was also found to be significant [*F*(3,36)=8.16, *P*=0.0045].

Next, we examined the effect of S961 on beta cell volume in the different-sized bins (Fig. 4D). In the small islet category, the beta cell volume was 0.26±0.01 mm^3^ in the vehicle group and 0.3±0.01 mm^3^ in the S961 group, representing a 15.3% increase in the S961 group. For the medium-sized islets, we detected a 50.5% increase in beta cell volume (*P*≤0.05), from 0.93±0.05 mm^3^ in the vehicle group to 1.4±0.1 mm^3^ in the S961 group. For the large islets, the beta cell volume was 1.19±0.07 mm^3^ in the vehicle group and 1.6±0.09 mm^3^ in the S961 group, corresponding to a 34.4% increase in the S961 group. Finally, for the very large islets, beta cell volume was 0.55±0.07 mm^3^ in the vehicle group and 0.9±0.15 mm^3^ in the S961 group, corresponding to a 97.8% increase in the S961 group. For the islet volume data, statistical analysis revealed significant main effects of both treatment [*F*(1,12)=15.87, *P*=0.0018] and size category [*F*(3,36)=86.28, *P*<0.001], but no significant two-way interaction was found [*F*(3,36)=3.39, *P*=0.0513]. As the islet count data and the beta cell volume data are correlated measures, pairwise post hoc tests on the treatment effect within each size category were carried out in a multivariate manner using Hotelling's T-squared test. The pairwise tests showed significant treatment effect for small (*P*=0.0233), medium (*P*=0.0094) and large islets (*P*=0.0121), but not for very large islets (*P*=0.1694).

### Proliferation of beta cells is the most likely explanation for increased beta cell volume in response to S961 treatment

**Fig. 5.**
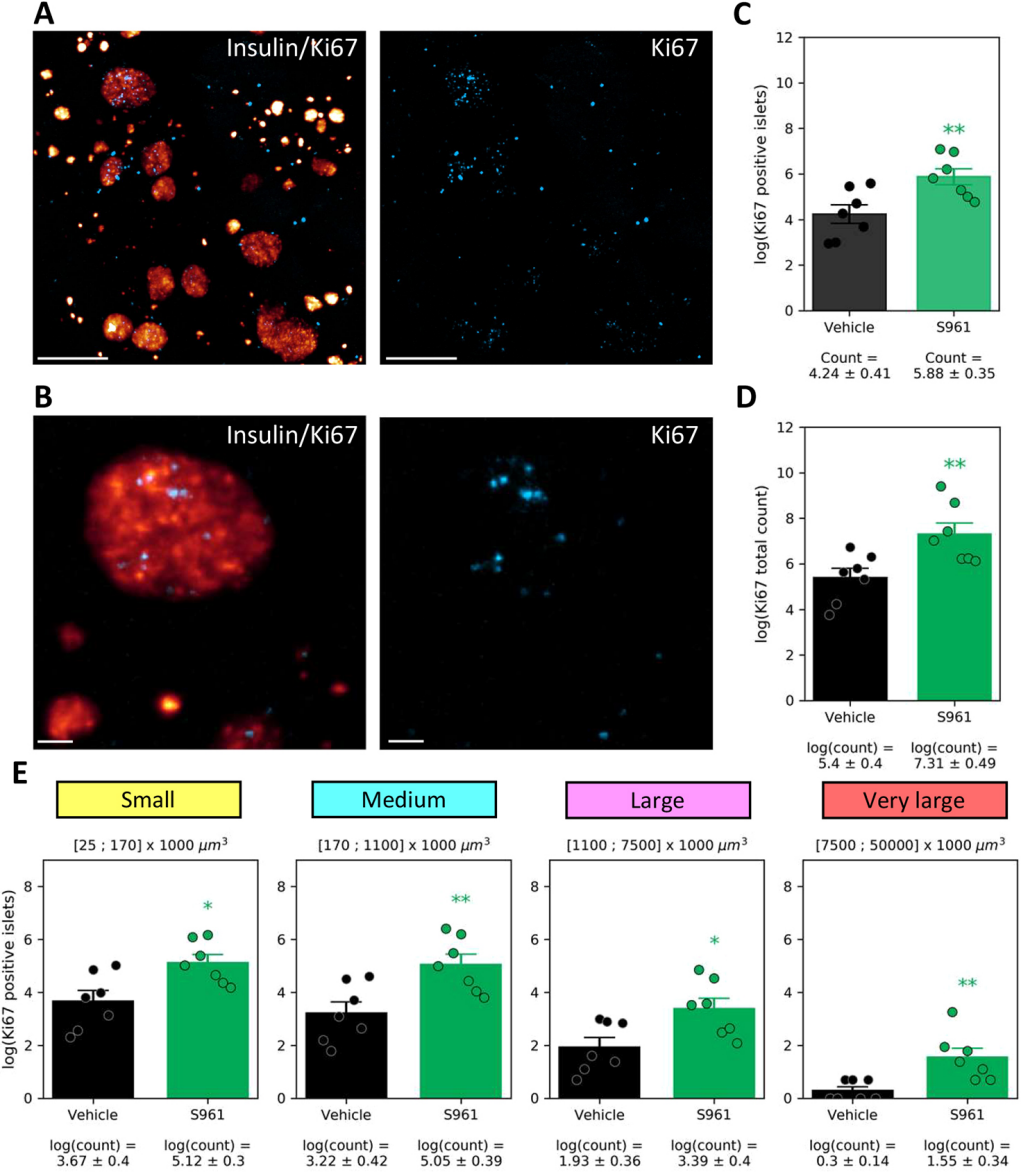
**Quantification of Ki67-positive beta cells.** (A) 3D light-sheet microscopy image from a S961-treated mouse showing insulin (glow scale) and Ki67 (blue) staining. The Ki67 channel is shown separately on the right. (B) High-magnification section (20 µm) from the 3D image stack, showing insulin and Ki67 staining in an islet. (C) Total number of Ki67-positive islets is increased in the S961 group in comparison to the vehicle group. (D) Total number of Ki67-positive cells within all segmented islets is increased in the S961 group in comparison to the vehicle group. (E) Quantification of the number of small (yellow), medium (cyan), large (magenta) and very large (red) insulin-positive islets with Ki67 signal in vehicle- and S961-treated mice. Individual data points are indicated. For pairwise statistical analysis (in C-E), unpaired Student's *t*-test was used. *P<0.05, **P<0.01 (*P*=0.0022 in E); S961 compared to vehicle. In E, mixed ANOVA was applied prior to the pairwise statistics. Error bars represent s.e.m. Scale bars: 400 µm (A); 100 µm (B).

By staining pancreata for Ki67, we set out to quantify the number of proliferating beta cells. Light-sheet microscopy enabled the identification of individual proliferating cells within 3D-imaged pancreata (Fig. 5A,B). Using insulin staining to segment the beta cell volume, we were able to quantify Ki67-positive nuclei within this volume. The analysis illustrates a significant increase in the total number of Ki67-positive islets and in the total number of proliferating cells within the beta cell volume across pancreata in the S961 group in comparison to the vehicle group (Fig. 5C,D). Quantification of Ki67 signal by islet size categories demonstrates that there was an increase in proliferation among islets of all analyzed sizes, albeit with variation between samples (Fig. 5E). In statistical analysis of the number of Ki67-positive islets, significant effects were found for both treatment [*F*(1,12)=10.92, *P*=0.0063] and size category [*F*(3,36)=180.62, *P*<0.001]. No significant two-way interaction was observed [*F*(3,36)=1.03, *P*=0.3590].

Unpaired Student's *t*-test were performed on the treatment effect within each size category, which indicated significance within all size categories: small (*P*=0.0129), medium (*P*=0.0072), large (*P*=0.0189) and very large (*P*=0.0051).

### Identification of early signs of type I diabetes in mouse using light-sheet imaging

**Fig. 6.**
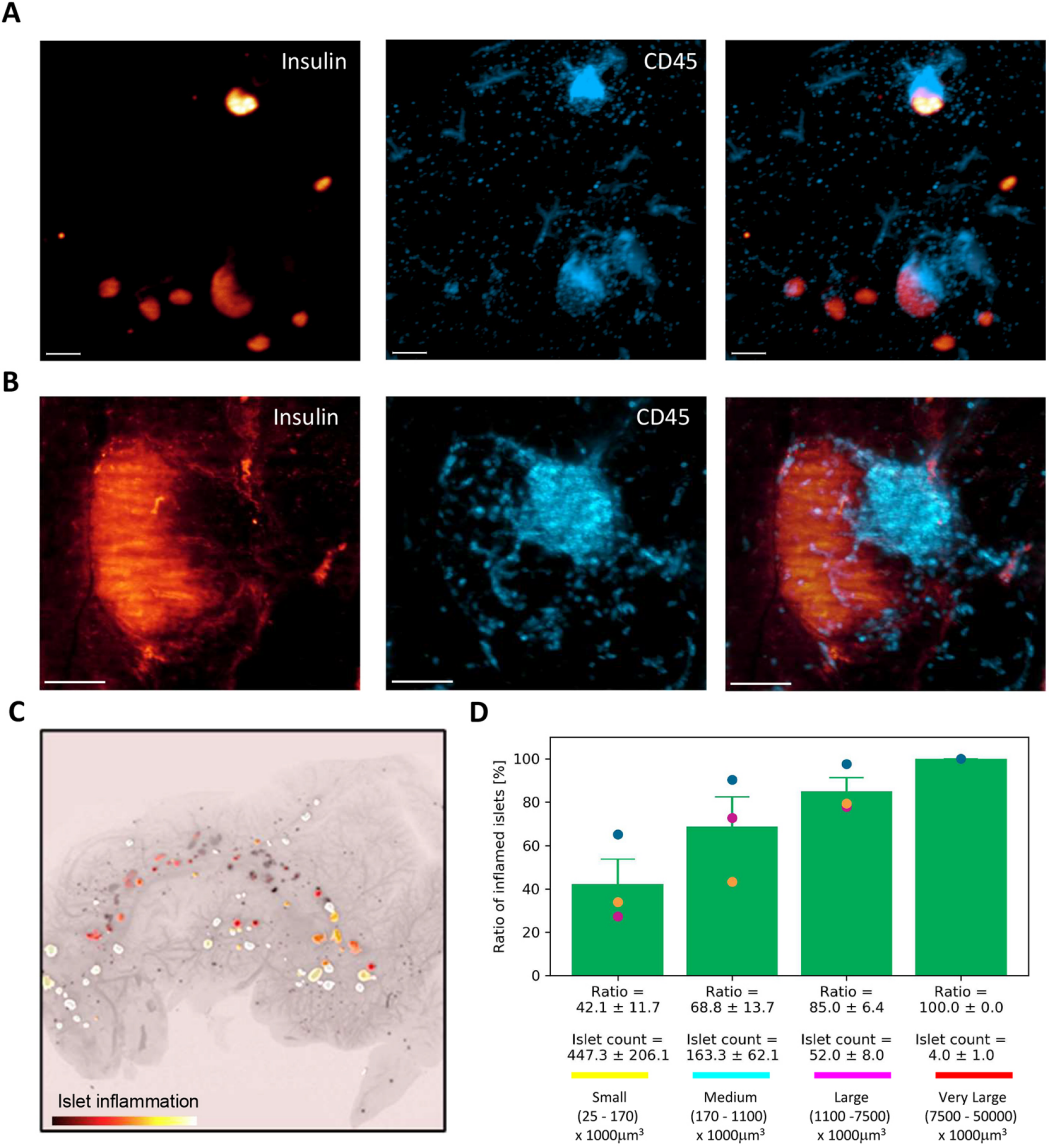
**3D imaging of islet inflammation in NOD mice.** (A) 3D light-sheet microscopy image of an area in 14-week-old NOD mouse pancreas, stained for insulin (glow scale) and CD45 (blue). Section from the 3D image stack, demonstrating insulin (glow scale) and CD45 (blue) staining in the 14-week-old NOD mouse. (B) High-magnification image of an individual islet from the NOD mouse, demonstrating insulin signal (glow scale) and leukocyte (CD45; blue) infiltration within the islet and accumulation at the islet periphery. (C) Overview image of a pancreas head, with islet inflammation segmentation data in glow scale overlaid with islet insulin segmentation data. Higher prevalence of CD45^+^ cells within an islet results in brighter islet color. (D) Quantification of the number of inflamed small (yellow), medium (cyan), large (magenta) and very large (red) insulin-positive islets. Islets with at least 5% volume of CD45^+^ cells were quantified. Individual data points from different mice are indicated in different colors. Error bars represent s.e.m. Scale bars: 200 µm (A); 100 µm (B).

Female NOD mice develop hyperglycemia at ∼14 weeks of age (males a few weeks later) (Mathews et al., 2015). The onset is variable, and, at 5 weeks, immune cell infiltrates can already be detected (DiLorenzo et al., 1998). We reasoned that 3D light-sheet imaging can capture the inflammatory changes in pre-diabetic NOD mice and thus analyzed pancreata from 14-week-old female mice. At the time of termination, blood glucose was normal (data not shown). The dissected pancreata were stained for insulin and the general leukocyte marker CD45 (also known as PTPRC) (Fig. 6A; Movie 3). Light-sheet microscopy enabled the visualization of leukocytes across pancreata, although in highly inflamed areas individual cells could not be distinguished as CD45 antigen protein tyrosine phosphatase receptor type C is a membrane protein (Fig. 6A,B). Using the islet segmentation platform as described above, we identified insulin-labeled islets, classified these into size categories and quantified CD45 staining within these as a fraction of total islet volume (Fig. 6C). A 5% volume threshold (CD45 signal from total islet volume) was implemented to define an inflamed islet. We observed that 42.1±11.7% of islets in the small islet category, 68.8±13.7% of islets in the medium islet category, 85±6.4% of islets in the large islet category and 100% of the islets in the very large islet category were positive for CD45 (Fig. 6D). These data demonstrate extensive islet infiltration in NOD mice at the onset of diabetes, whereby a large majority of medium- to large-sized islets are already inflamed.

## DISCUSSION

The aim of this study was to demonstrate a method for accurate measurement of islet beta cell volume, proliferation and inflammation in mice using light-sheet microscopy and automated 3D image analysis. In particular, 3D imaging of entire pancreata enabled us to reveal the changes in different islet size categories. Using the herein developed methods, we show that, in a mouse model of S961-induced hyperglycemia and impaired insulin sensitivity, there is an overall increase in beta cell volume and islet number. In the NOD mouse model of type I diabetes, we show that, prior to the onset of hyperglycemia, more than 80% of large islets are already infiltrated by leukocytes.

A particular strength of 3D light-sheet imaging is the capacity to carry out volumetric analysis of islets in different size categories and to characterize various parameters within these. Heterogeneity of islets and functional differences in small and large islets are well established (Aizawa et al., 2001; Baetens et al., 1979; Huang et al., 2011; Roscioni et al., 2016). It is, for example, conceivable that different sources of beta cell regeneration may impact islet size distribution differently, and accurate analysis of the process is needed for preclinical drug efficacy studies. Neogenesis or trans-differentiation may increase the number and volume of small islets more than those of large islets, while general beta cell proliferation may result in a more uniform response across size categories.

To study the response to acquired impairment in insulin sensitivity, we used the insulin receptor antagonist S961. Chronic dosing in male high-fat-diet-induced obese mice resulted in increased levels of insulin and blood glucose, indicating that the S961 dose chosen was sufficient to drive a sustained physiological response. Our 3D image analysis demonstrated an overall increase in beta cell volume and in islet count. Analysis of the response in islet size categories showed an increase in both the number and volume of small-, medium- and large-sized islets in the S961-treated group. The number of islets and their mean volume is well known to depend on disease phase in patients with type II diabetes. An early compensatory hypertrophy of islets is later followed by beta cell loss (Chen et al., 2017). In support of this, the presented Ki67 analysis showed an overall increased labeling in the S961 group, suggesting higher beta cell proliferation. This result corresponds with previous studies showing that beta cell proliferation is the main factor driving the expansion of beta cell volume in response to S961 dosing (Dumayne et al., 2020; Jiao et al., 2014; Yi et al., 2013). Similarly, increased islet number in S961-treated mice has also been observed by other groups (González-Mariscal et al., 2018; Okamoto et al., 2017). However, these results do not rule out alternative mechanisms resulting in increased beta cell count and volume. These include trans-differentiation of other cell types into beta cells and islet neogenesis. Other islet cell types (Ben-Othman et al., 2017; Cigliola et al., 2018; Li et al., 2017; Zhou et al., 2008) and ductal epithelium (Bonner-Weir et al., 2012) may contribute to insulin-producing cells. More detailed analysis of different cell types in early phases of S961 treatment could provide clues into biological processes in early type II diabetes.

Type I diabetes results from autoimmune destruction of beta cells. The NOD mouse model has provided valuable insights into the disease progression, but direct translation to new therapeutics has been challenging (Pearson et al., 2016; Reed and Herold, 2015; Sandor et al., 2019). High variability, both in NOD mice and in patients, and still inadequately understood mechanisms of the immune response have hindered pharmacological advances. Here, we established a light-sheet imaging platform to characterize immune cell infiltration in islets in the NOD model. We show that, before any changes in blood glucose can be detected, 80% of large islets and 40% of small islets are infiltrated by CD45^+^ immune cells. Previous intravital imaging studies, using fluorescently labeled immune cell subsets have established the early onset of the disease at 3-5 weeks of age, whereby stochastic homing and infiltration of islets occur at first by individual infiltration of autoreactive T-cells (Coppieters et al., 2012; Lindsay et al., 2015; Mohan et al., 2017). This early infiltration occurs significantly prior to the development of hyperglycemia at 14 weeks (Mathews et al., 2015). After the initial infiltration of the islets, they are targeted by multiple leukocyte subtypes from the islet periphery (Mohan et al., 2017). In support of this, we found accumulation of leukocytes in the islet periphery at 14 weeks. The light-sheet imaging platform demonstrated in the present study does not rely on transgenic reporters or *in vitro* cell labeling and is particularly amenable to pharmacological research in type I diabetes. Additional antibodies for immune cell subsets can be included, enabling the definition of changes in the immune cell repertoire.

In conclusion, we demonstrate here a 3D imaging platform for the analysis of changes in entire pancreata in mouse models of type I and II diabetes. We show that the insulin receptor antagonist S961 leads to increased islet number and volume, likely due to proliferative response within beta cells. In the NOD mouse model of type I diabetes, we found that, prior to the onset of hyperglycemia, the vast majority of islets already show significant leukocyte infiltration. The established methodology can be adjusted to incorporate additional markers of interest (i.e. activated cell signaling pathways, leukocyte subsets, drug target receptors) for gaining better understanding of the basic biological mechanisms of type I and II diabetes and for pharmacological analysis of the efficacy of therapeutics.

## MATERIALS AND METHODS

### Animals

Male C57Bl/6J mice were obtained from Janvier Labs (Le Genest-Saint-Isle, France) and maintained in standard housing conditions (12 h light/dark cycle and controlled temperature of 21-23°C). Mice had *ad libitum* access to tap water and 60% high-fat diet (high-fat Ssniff diet D12492; Brogaarden, Hørsholm, Denmark; 60% kcal% fat (91% lard, 9% soybean oil), 20% kcal% protein (98.5% casein, 1.5% L-cystine), 20% kcal% carbohydrate (63% Lodex-10, 37% sucrose). Groups were age matched (30 weeks). When the mice reached a body weight of ∼50 g, they were randomized into two groups according to body weight (*n*=7). Alzet osmotic minipumps (1002; Alzet, Cupertino, CA, USA) were implanted, containing either vehicle (PBS) or S961 peptide (Schafer-N, Copenhagen, Denmark) dissolved in PBS at a 0.4 nmol/µl concentration for 14 days. According to the manufacturer, this corresponds to a daily release of 2.4 nmol/kg. NOD/ShiLtJ female mice were purchased from Charles River Laboratories and maintained on regular chow (Altromin 1324; Brogaarden) and were terminated at 14 weeks. All animal procedures were conducted in compliance with internationally accepted principles for the care and use of laboratory animals and were approved by the Danish Animal Experiments Inspectorate (license 2013-15-2934-00784).

### *In vivo* measurements

For blood glucose measurement, blood samples were collected into heparinized glass capillary tubes and immediately suspended in glucose/lactate system solution buffer (EKF-Diagnostics, Barleben, Germany). Blood glucose was measured using a BIOSEN c-Line glucose meter (EKF-Diagnostics), according to the manufacturer's instructions. For insulin measurement, blood samples were similarly collected in heparinized tubes and plasma was separated and stored at −80°C until analysis. Mouse insulin was measured using the Meso Scale Diagnostics platform.

### Sample preparation for immunohistochemistry

Animals were transcardially perfused with heparinized PBS and 40 ml of 10% neutral buffered formalin (CellPath, Newtown, UK) under Hypnorm-Dormicum (fentanyl 788 µg/kg, fluanisone 25 mg/kg and midazolam 12.5 mg/kg, subcutaneous injection) anesthesia. Pancreata were carefully dissected and immersion fixed in 10% neutral buffered formalin overnight at room temperature on a horizontal shaker. The samples were washed 3×30 min in PBS with shaking and dehydrated at room temperature in methanol/H_2_O gradient to 100% methanol (20%, 40%, 60%, 80%, 100% methanol; each step 1 h). The pancreata were stored in 100% methanol (VWR International A/S, Søborg, Denmark) at 4°C until further processing.

### Whole-pancreas immunohistochemistry for labeling of insulin and Ki67

For whole-pancreas immunohistochemistry, a modified version of the original iDISCO (immunolabeling-enabled three-dimensional imaging of solvent-cleared organs) protocol was used (Renier et al., 2014, 2016). Samples were washed with 100% methanol for 1 h and incubated overnight in 66% dichloromethane/33% methanol (VWR International A/S) at room temperature. Then, samples were washed twice in 100% methanol for 30 min and bleached in chilled fresh 5% H_2_O_2_ (Acros Organics, Fisher Scientific Biotech Line A/S, Slangerup, Denmark) in methanol overnight at 4°C. Subsequently, the samples were rehydrated in methanol/PBS series [80%, 60%, 40%, 20% methanol with 0.2% Triton X-100 (Merck, Darmstadt, Germany); each step 1 h] at room temperature, washed in PBS with 0.2% Triton X-100 twice for 1 h at room temperature and in permeabilization solution [PBS with 0.2% Triton X-100, supplemented with 20% volume dimethyl sulfoxide (DMSO; Merck, Darmstadt, Germany) and 2.3% weight/volume glycine (Merck)] for 3 days at 37°C. Unspecific antibody binding was blocked by a 2-day incubation in blocking solution [PBS, 2% Triton X-100, 10% DMSO/6% donkey serum (Jackson ImmunoResearch, Cambridgeshire, UK)]. Immunohistochemistry was carried out sequentially, by first incubating the samples for 7 days at 37°C with anti-Ki67 antibody (1:200 dilution; NB110-89717; Novus Biologicals, Centennial, CO, USA) or with anti-CD45 (1:200 dilution; 550539; BD Pharmingen, CA, USA), diluted in PTwH [PBS, 0.2% Tween 20 (Merck), 0.1% of 10 mg/ml heparin solution], 5% DMSO, 3% donkey serum, 0.2% of 10% NaN_3_ (Merck). Following incubation with primary antibody, the samples were washed in PTwH for 1×10 min, 1×20 min, 1×30 min, 1×1 h, 1×2 h and 1×2 days. Subsequently, the pancreata were incubated in secondary antibody solution (PTwH, 3% donkey serum, 0.2% of 10% NaN3) for 7 days at 37°C with Alexa Fluor 790 AffiniPure Donkey Anti-Rabbit IgG (1:1000 dilution; 711-655-152; Jackson ImmunoResearch) or Anti-Rat-Cy3 IgG (1:1000 dilution; 712-165-153; Jackson ImmunoResearch) and washed in PTwH for 1×10 min, 1×20 min, 1×30 min, 1×1 h, 1×2 h and 1×3 days. Samples were post-fixed in 10% neutral buffered formalin overnight and incubated in Alexa Fluor 647-conjugated anti-insulin antibody (1:500 dilution; 9008; Cell Signaling Technology, Danvers, MA, USA) in the above-described antibody dilution buffer. The samples were subsequently washed again in PTwH for 1×10 min, 1×20 min, 1×30 min, 1×1 h, 1×2 h and 1×3 days and embedded in 1% low-melting-point agarose (16520050; Thermo Fisher Scientific; dissolved in PBS). The agarose-embedded pancreata were dehydrated in increasing concentrations of methanol (20%, 40%, 60%, 80%, 100%; 1 h each at room temperature), followed by overnight incubation in 100% methanol. Samples were next incubated in 66% dichloromethane/33% methanol for 3 h at room temperature with shaking and in 100% dichloromethane twice for 15 min with shaking to remove traces of methanol. Finally, the samples were transferred to dibenzyl ether (Merck) and stored in closed glass vials until imaging with light-sheet fluorescence microscopy.

### Light-sheet fluorescence microscopy of cleared immunolabeled pancreata

All agarose-embedded pancreata were imaged on a LaVision ultramicroscope II setup (Miltenyi Biotec, Bergisch Gladbach, Germany) equipped with a Zyla 4.2P-CL10 sCMOS camera (Andor Technology, Belfast, UK), SuperK EXTREME supercontinuum white-light laser EXR-15 (NKT Photonics, Birkerød, Denmark) and MV PLAPO 2XC (Olympus, Tokyo, Japan) objective lens. The samples were attached to the sample holder with neutral silicone and imaged in a chamber filled with dibenzyl ether. Version 7 of the Imspector microscope controller software was used. Images were acquired at 0.63× magnification (1.2× total magnification) with an exposure time of 266 ms for insulin and 1 s for Ki67 in a *z*-stack at 10-µm intervals. Acquired volumes (16-bit tiff) had an in-plane resolution of 4.8 µm and *z*-resolution of 3.78 µm (NA=0.156). High numerical aperture is needed to capture individual Ki67^+^ cell nuclei; however, this results in uneven light-sheet thickness. To alleviate the effect of this, horizontal focusing was captured in nine planes with contrast-based blending of the images.

### Image processing for insulin and CD45 segmentation

U-Net network architecture (Ronneberger et al., 2015) was used to create a 2D U-Net with four repeated layers for encoding and four repeated layers for decoding, implemented in Python utilizing the Keras machine learning library (https://github.com/keras-team/keras). The U-Net input was a single intensity channel and the output was a single label image. Raw images were downsampled by a factor of 2 to a size of 1024×1024 pixels. Annotations were performed manually on a total of 154 image tiles with a size of 512×512 pixels. Intensities of the training images were rescaled between 0 and 1; 75% of the data were used for training, 20% for validation and 5% for testing. Data augmentation, in the form of skews, rotations, flips, zoom and random distortions, was applied during training with probability of 30% for each operation. Training was performed for 350 epochs and the model achieved a dice coefficient of 0.79 on the validation set. The trained model was afterwards used to segment full-size 2048×2048 pixel images. For an example of the segmentation see Fig. S2.

### Quantification of Ki67-positive cells

Background subtraction through morphological opening using a disk element was performed slice-by-slice on raw Ki67 intensity images. To identify Ki67-positive cell candidates, local intensity peaks were located by moving a filter cube [5×5×3 (*x*,*y*,*z*) voxels] over the image volume. The coordinates of detected local intensity peak candidates were used as seeds in a watershed segmentation with a background intensity cut-off at 50, and the resulting segmentations were filtered by removing cell segmentation regions smaller than 4 voxels and bigger than 100 voxels.

### Statistics

Pairwise treatment effects were investigated using unpaired Student's *t*-tests or Hotelling's T-squared test. Additionally, in multiple cases, the treatment effect was investigated specifically for different islet size categories. This required binning the data into four different size categories. As the binnings were splits of the full data set, this introduced a correlated factor to the analysis. Thereby, data were analyzed as a two-way 2×4 (two treatments, four size categories) mixed ANOVA, with the treatment as a between-subjects factor and the size category as a within-subjects factor. For all mixed ANOVAs, the Greenhouse–Geisser correction was used for *P*-values related to the within-subjects factor, if Mauchly's test of sphericity indicated that the assumption of sphericity was violated. All statistics were carried out using R (https://www.r-project.org/), and statistical results are commonly presented alongside mean±s.e.m.

## Supplementary Material

Supplementary information
