# Supplementary material for: 3D quantification of changes in pancreatic islets in mouse models of diabetes type I and II
Source: Dis Model Mech. 2020 Dec 18;13(12):dmm045351. doi: 10.1242/dmm.045351 (PMC7758639; doi:10.1242/dmm.045351)
Supplement: Supplementary information [file dmm-13-045351-s1.pdf]

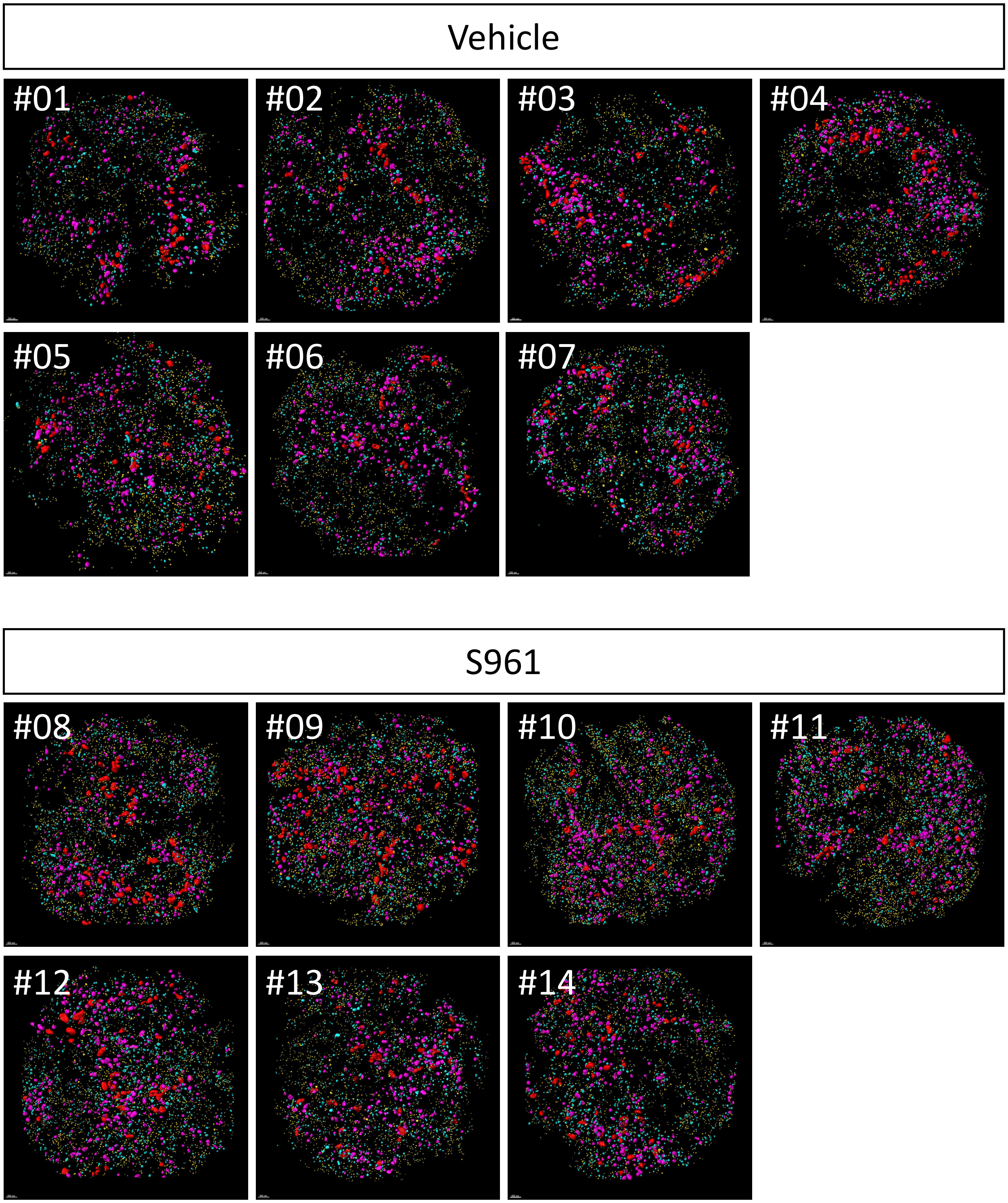

Figure S1. Overview of all pancreata in the two groups: For size distribution the insulin positive islets were allocated into four bins, small (yellow; 25-170 x1000 μm^3), medium (cyan; 170-1100 x1000μm^3), Large; (magenta; 1100-7500 x1000μm^3) and very large (red; 7500-50000 x1000μm^3).

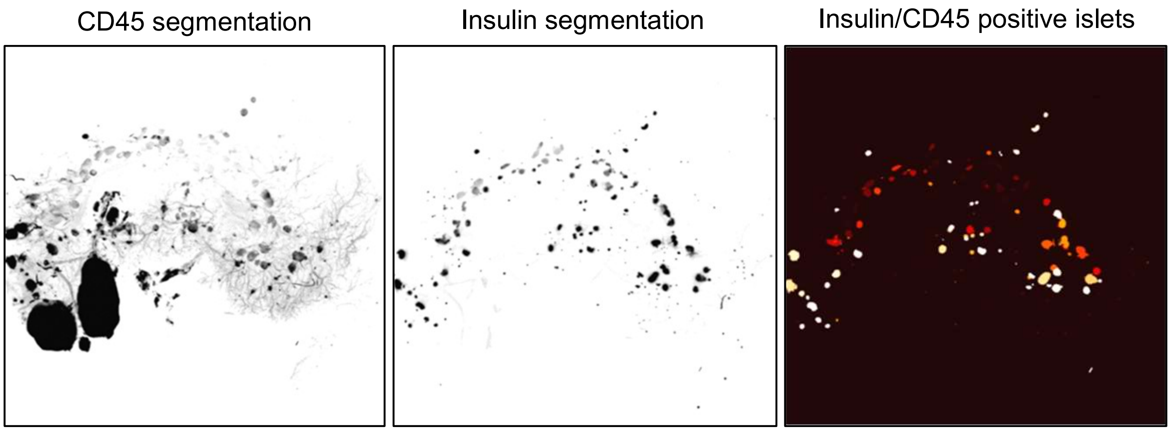

Figure S2. Computational identification of inflamed islets: Computational identification of CD45 signal (in greyscale, note that large black areas represent lymph nodes), insulin signal in the middle panel and on the right, identification of insulin positive islets in 3D imaged pancreas with CD45 signal.

Disease Models & Mechanisms • Supplementary information

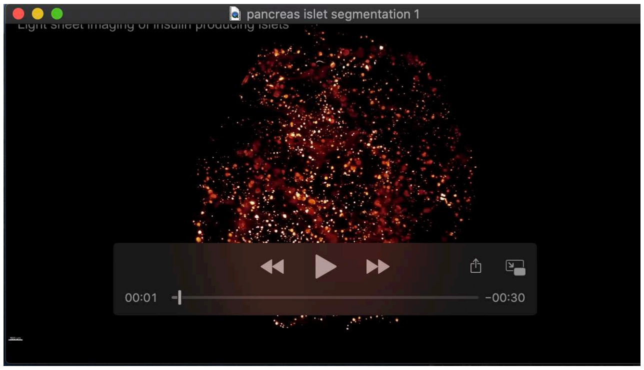

Movie 1: Segmentation of the insulin signal: the segmentation of individual islets based on insulin signal (cyan).

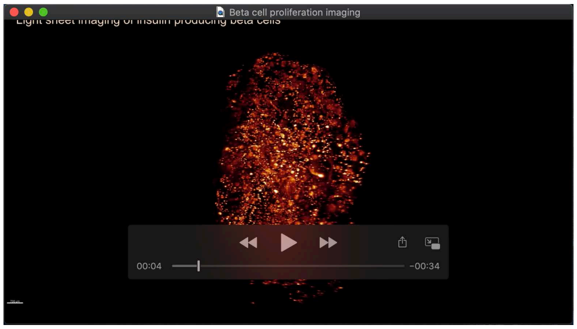

Movie 2: Mouse pancreas immunolabelled with insulin and Ki67: A whole mouse pancreas from a S961 dosed mouse labelled with antibodies against insulin (cyan) and Ki67 (glow scale).

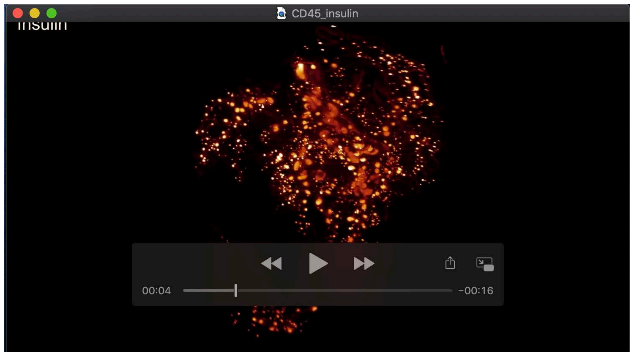

Movie 3: Double labelling of NOD pancreas: Movie showing the infiltration of pancreatic islets in a NOD mouse. The pancreas was stained for insulin (cyan) and CD45 (glow scale)
